# Virginia State Veterinary Medical Association

**Published:** 1895-02

**Authors:** Geo. C. Faville

**Affiliations:** Secretary


Secretary.

### VIRGINIA STATE VETERINARY MEDICAL ASSOCIATION.

THE third regular meeting was called to order in the old Bank Building, in Charlottesville, Virginia, January 3, 1895, at 10 o'clock A.M., by the President, Dr. W. H. Harbaugh, of Richmond,

The following members answered at roll-call: Dr. Harbaugh, of Richmond; Dr. T. A. Donaldson, of Richmond; Dr. E. P. Niles, of Blacksburg; Dr. W. T. Gilchrist, of Norfolk; Dr. Geo. C. Faville, Norfolk; Dr. C. P. Dixon, of Charlottesville; Dr. H. Marshall, of Charlottesville; Dr. D. B. Miller, of Charlottesville. Visitors present were Drs. J. E. Miller, of Ironton, Ohio, and C. B. Robinson, of Washington, D. C.

After reading the minutes of the last meeting the report of the Board of Censors was called for, and from them the following were proposed for admission to membership and were elected: Dr. F. P. Williamson, Raleigh, N. C.; Dr. R. D. Garcen, Richmond, Va.; Dr. Chas. McCulloch, Jr., Howardsville, Va.; Dr. Elias S. Walmer, Washington, D. C.; Dr. John E. Miller, Richmond Va; Dr. C. Barnwell Robinson, Washington, D. C.

Dr. Niles, chairman of the Committee on Statistics, while making no report, made a request of the members that they should report to him upon blanks furnished upon cases of interest, especially those bearing upon heredity.

Under the head of new business, Dr. Harbaugh called attention to the following resolutions which was adopted by the U. S. V. M. A. at its last meeting: "*Whereas*, We, the United States Veterinary Medical Association, do condemn the operation of docking horses' tails as an operation of fashion, believing that nature intended them as a defence against insects, etc.; but, so long as it is demanded as an operation of fashion, we believe that it should be performed by skilled veterinarians, who are sure to perform it with greater care and less pain and suffering to the animal, and we cannot unqualifiedly condemn those who perform the operation under such conditions." Dr. Harbaugh could not see the consistency of the resolution, in one part condemning the operation and in the next condoning the operator. Dr. Gilchrist introduced the following resolutions, which were adopted: *Whereas*, The horse's tail is intended by nature as a defence against pestiferous insects, etc., the Virginia State Veterinary Medical Association unqualifiedly condemns the practice of docking tails for mere fashion's sake. There are rare instances when it is necessary to amputate a portion of the tail on account of disease or injury, as it is often necessary to amputate an arm or a leg of a human being, but the indiscriminate habit of docking tails serves no useful purpose whatever, and entails needless suffering on the part of a helpless dumb brute, not only from the manner in which the operation is usually performed, but also during the remainder of the animal's life from various insects; therefore, be it *Resolved*, That we, the members of the Virginia State Veterinary Medical Association, pronounce the docking-shears an instrument of inhuman contrivance, and consider the habit of docking tails a cruel and barbarous practice. *Resolved*, That we do not consider the unnecessary docking of tails a surgical operation, and members of this Association are justified in refusing to perform it. After an animated discussion of the question the resolution was adopted.

Dr. Adamson, who was appointed to read a paper upon "Rabies," was not present. His paper was read by the Secretary, and elicited considerable discussion, after which the society adjourned to partake of a substantial lunch, a compliment from the members resident in Charlottesville.

Promptly at four o'clock the association reassembled and listened to some very interesting notes by Dr. Niles, which were really a continuation of his paper on "Tuberculosis," read at the August meeting. The recent experiments by Dr. Niles with the use of tuberculin were of great interest to all, and elicited a warm discussion.

Drs. Faville and Gilchrist reported a case of "Encephalitis," which was listened to with interest, after which Dr. Harbaugh, of Richmond, read a very interesting paper upon "Texas Fever," illustrating his points by copious notes of cases which he cited. This paper drew out considerable discussion, particularly because of the difference of opinion expressed as compared with the results of the experiments and conclusions of the Bureau of Animal Industry. Dr. Harbaugh cited many cases which seem to disprove the "tick theory" of the bureau. After a recess taken for supper the association was reconvened in the office of Drs. Marshall and Dixon, and enjoyed a general discussion. Dr. Niles reviewed informally his experience as veterinary inspector for the Virginia State Exposition. From his experience it would seem as though veterinary inspection was not greatly appreciated in the State, and it is doubtful whether any member of the Association would run the risk of similar treatment by undertaking to examine any animals for the Exposition in the future.

After a general discussion upon the needs of advanced education and educational institutions in this country, especially as regards *entrance* and final examinations, the association adjourned to hold its annual meeting at the Agricultural College on the fourth Wednesday in June.

GEO. C. FAVILLE, D.V.M.,
Secretary.

